# Source strength assay of iodine‐125 seeds sealed within sterile packaging

**DOI:** 10.1120/jacmp.v14i2.4082

**Published:** 2013-03-04

**Authors:** Yuki Otani, Takahiro Yamada, Shingo Kato, Naoto Shikama, Kazuto Funakoshi, Isao Kuroda, Hodaka Numasaki, Takayuki Nose, Takushi Dokiya, Masahiko Oguchi

**Affiliations:** ^1^ Department of Radiation Oncology Osaka University Graduate School of Medicine Suita Osaka; ^2^ Japan Radioisotope Association Tokyo; ^3^ Department of Radiation Oncology Saitama Medical University Saitama; ^4^ Department of Uro‐oncology Saitama Medical University Saitama; ^5^ Department of Medical Physics and Engineering Osaka University Graduate School of Medicine Osaka; ^6^ Department of Radiation Oncology Nippon Medical School, Tamanagayama Hospital Tokyo; ^7^ Department of Radiation Oncology Cancer Institute Hospital, the Japanese Foundation for Cancer Research Tokyo Japan

**Keywords:** batch assay, I‐125 seeds, permanent implant brachytherapy, prostate cancer, sterile convenience pack

## Abstract

Early‐stage prostate cancer is widely treated by iodine‐125 (I‐125) seed implantation. While quality assurance methods are in place to assure consistency in I‐125 seed source strength, current methods involve the breaking of the sterilization package, raising issues concerning sterility and time limitations. The purpose of this study was to develop a method of characterizing the total source strength of I‐125 seeds within a cartridge that has been sealed within a sterilization package and to evaluate the probability of detecting an out‐of‐calibration seed (aberrant seed). We defined a protocol to determine the ability of a well‐type ionization chamber to detect aberrant I‐125 seeds within a cartridge sealed in the sterilization package. A novel jig for a well‐type ionization chamber was designed to accommodate the sterilization package. One seed was chosen randomly from two cartridges containing five or 15 seeds (0.544 U source strength) and was exchanged with aberrant seeds of six different source strengths. The source strength was measured at each position within the cartridge. The results indicated that the response of the well chamber was sensitive to changes in the aberrant seed position within the cartridge and the source strength of the aberrant seed. The correlation coefficient between single seed and batch assay results was high (0.998). A novel jig and a measurement method using a well ionization chamber were developed, which allowed for a batch assay characterization of the total source strength of I‐125 seeds within a cartridge sealed within sterilization package. This method is simple, time‐saving, and offers greater practical application.

PACS number: D6.20.Dk

## I. INTRODUCTION

Iodine‐125 (I‐125) permanent implant brachytherapy is a widely used modality in the treatment of prostate cancer.^(^ ^1^ ^–^ ^5^ ^)^ Between 2003, when prostate brachytherapy was first introduced as a form of treatment in Japan, and 2010, a total of 15,427 patients were treated at 107 Japanese institutions.^(^ ^6^ ^)^ The OncoSeed model 6711 (General Electric Healthcare, Barrington, IL) is the type of therapeutic I‐125 seed used by most Japanese institutions and is delivered in a sterile convenient blister pack (SCBP). Currently used only in Japan, this form of SCBP, which contains a preloaded five‐seed or 15‐seed cartridge, was jointly developed by two companies (General Electric Healthcare and Nihon Medi‐Physics, Tokyo, Japan) as an improvement on the conventional sterile convenient Tyvek pack (SCTP) (DuPont, Wilmington, DE) with respect to prevention of seed loss from the cartridge and damage to the sterilization package.

The American Association of Physicists in Medicine (AAPM) Task Group 64 recommended that institutional medical physicists perform seed calibration on at least 10% of the seeds in all shipments to satisfy the need for an independent assay of a random sample.^(^ ^7^ ^)^

However, there are several downsides to the evaluation of individual seeds. It is extremely time‐consuming, involves exposing hospital personnel to frequent doses of radiation, and poses a challenge regarding the resterilization procedure. To meet these challenges, several batch assay methods have been developed, among which the two main approaches are the source holder approach and the cartridge approach. In the source holder approach, several seeds are removed from the cartridge and loaded in a source holder to allow the measurement of their source strength, typically using a well ionization chamber.^(^ ^8^ ^–^ ^10^ ^)^ This method can assess the source strength of various seeds with one measurement, but the operators must return the seeds to the cartridge after the assay. The cartridge approach can assess the source strength using an imaging plate or a well ionization chamber, with the seeds still in the cartridge.^(^ ^11^ ^–^ ^12^ ^)^ Although the cartridge approach can effectively assess the source strength while preserving sterilization, it requires the sterile inserts to be deployed in the operating room relatively close to the time of implant and, hence, poses the risk of seeds being lost and inserts being contaminated. These disadvantages have encouraged the development of a new method of characterizing the total actual source strength of I‐125 seeds within a cartridge sealed in an SCBP and of evaluating the probability of detecting aberrant seeds. The purpose of this study was to develop a method that achieves these goals in a time‐efficient manner without encountering the disadvantages of the other methods of measurement currently in use.

## II. MATERIALS AND METHODS

### A. Dosimetry methods and materials

**Figure 1:**
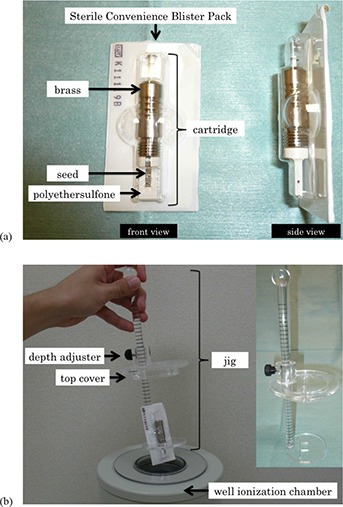
Photographs showing (a) the front and side of the SCBP and (b) the novel jig specifically developed for use in this study.

A well ionization chamber (Capintec Inc., Ramsey, NJ; CRC‐15BT Dose Calibrator) calibration standard for the OncoSeed model 6711 was obtained from the Japan Radioisotope Association (calibration uncertainty was 6.0% k=2). The common single‐seed assay was used to confirm the manufacturer's claims that the source strength among seeds varied by less than 5% (Table 1). Figure 1(a) shows the appearance of SCBP. The front of the SCBP is covered with plastic and the back becomes the water‐repellent machined cardboard. A batch assay method was performed using a novel jig, developed by the authors, to stabilize and hold the SCBP (Fig. 1(b)). The jig was made of transparent acrylic material and constructed with a depth adjuster. It was designed in such a manner that the seed cartridge was centered in the well ionization chamber. The top cover of the jig was therefore designed to have a double structure so that the jig would fit firmly into the well ionization chamber.

**Table 1:** Differences between the nominal value and actual value for each air‐kerma strength.

*Air‐kerma Strength (U)*			*Difference Between Nominal Value and Measurements Value (%)*
	*Measurement Value*		
*Nominal Value*	*Mean (Range)*	*SD*	*Mean (Range)*
0.544	0.543 (0.522–0.556)	0.009	−0.20(−4.05–2.25)
0.460	0.461 (0.450–0.467)	0.005	0.14(−2.26–1.60)
0.434	0.435 (0.420–0.450)	0.008	0.16(−3.14–3.59)
0.395	0.401 (0.391–0.413)	0.005	1.60(−0.97–4.49)
0.367	0.377 (0.366–0.385)	0.005	2.73(−0.33–4.85)
0.336	0.336 (0.326–0.347)	0.006	0.23(−2.57–3.50)
0.310	0.313 (0.304–0.323)	0.005	1.44(−1.77–4.40)
0.285	0.285 (0.281–0.295)	0.003	−0.03(−1.52–3.38)

SD=standard deviation.

### B. Optimization protocol for the well‐type ionization chamber

Two basic experiments were performed before evaluating the batch assay. The first was designed to determine the optimal measurement depth of the batch assay because certain well chambers are known to exhibit a depth‐dependent response.^(^ ^13^ ^)^ The second experiment established that the measured source strength was linearly dependent on the number of seeds.

In this study, the shielded cartridge often used to reduce the radiation exposure of the operator was used. The shielded cartridge was made of brass, while the upper and the lower parts of the cartridge were made of polyethersulfone. Therefore, the response of the well chamber was potentially influenced by the presence of the brass in close proximity to the seeds.

### C. Position of the sample within the chamber

Two cartridges, one containing five seeds and the other containing 15 seeds within the SCBP, were set on the jig. The SCBP was set to a nominal depth of 0 cm, so that the top of the SCBP was touching the top cover of the jig. It was then lowered 7 cm in 0.5 cm increments, with a measurement acquired at each increment. The process was repeated three times.

### D. Rate of diminution ratio within the cartridge

The 17 seeds, with a source strength of 0.434 U, were loaded in a cartridge and numbered in increasing order such that the seed at the bottom of the cartridge was labeled No. 1. The cartridge was then repackaged in the SCBP and attached to the jig in preparation for measurement using the well ionization chamber. A series of measurements was performed by reducing the number of seeds in the cartridge one by one. The measurement was repeated three times and before each repetition, the seeds in the cartridge were rearranged randomly.

### E. Correlation between single‐seed and batch assays

To determine whether the batch assay on an intact SCBP reproduced the standard single‐seed assay, we calculated the correlation factor between the source strength of five‐seed and 15‐seed cartridges with single seeds. Eight different source strength seeds (source strength: 0.544, 0.460, 0.434, 0.395, 0.367, 0.336, 0.310, and 0.285 U) were evaluated to determine the correlation between the single‐seed and batch assays, with the correlation for the five‐seed and 15‐seed cartridges being evaluated separately. The result of the single‐seed assay was determined from the mean value of 15 seeds measured three times, while that of the batch assay was determined from the mean value of one batch measured three times. In the batch assay, after measurement of a 15‐seed cartridge, ten seeds were pushed out from the cartridge, a five‐seed cartridge was measured, and the seeds were exchanged and arranged randomly for every measurement.

### F. Detection sensitivity of aberrant I‐125 seeds

To investigate the impact of inclusion of an aberrant seed in the SCBP, one seed was chosen at random from two cartridges containing five or 15 seeds and exchanged. A seed with source strength of 0.544 U was used as the reference seed, and six seeds with different source strengths (source strength: 0.395 (73% of the 0.544 U), 0.367 (68%), 0.336 (62%), 0.310 (57%), 0.285 (52%), and 0 (0%) U) were used as the aberrant seeds. The position of each aberrant seed was interchanged at every possible position within the cartridge, and the source strength was measured at each position. The change in position of the aberrant seed was achieved by refilling the top of a cartridge seed pushed out from under a cartridge. Similarly, one seed was randomly selected from the reference seeds for measurement of its source strength when the position was changed.

### G. Clinical application of the batch assay

To evaluate the clinical relevance of the proposed methodology, the batch assay method was used for source strength measurements at our facility. Measurements of 15 cartridges containing five seeds and 69 cartridges containing 15 seeds were made on a weekly basis on the same day. In our facility, the nominal manufacturer's source strength of 0.460 U is always used.

## III. RESULTS

### A. Impact of cartridge position on measured source strength

**Figure 2:**
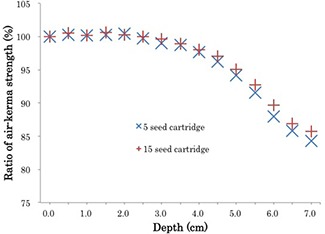
Change in measured source strength with change in depth in the well ionization chamber. The data were normalized with reference to the measured level at a depth of 0 cm. The limit of the top cover of the jig, to which the SCBP could be mounted without interference, was calculated to be a depth of 0 cm.

Figure 2 shows the changes in measured source strength as a function of depth normalized to a depth of 0 cm. The well chamber displayed sensitivity to the axial position in the signal output for a given source. Although no significant change from the source strength measurement at 0 cm was found at a depth of 2.5 cm, the measured source strength consistently decreased at depths beyond 2.5 cm, until it reached 84.3% and 85.7% of the original source strength at a depth of 7 cm in the five‐seed and 15‐seed cartridges, respectively. Based on this result, measurements were performed at a depth of 2 cm. The mean difference in measured source strength at 2 cm depth, as determined by the evaluation of three measurements, was found to be 0.6% (range: 0.2% to 1.0%) in the five‐seed cartridge and 0.3% (range: 0% to 0.7%) in the 15‐seed cartridge.

### B. Impact of seed number on cartridge measured source strength

**Figure 3:**
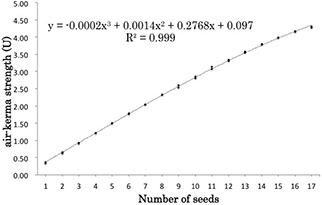
Change in measured source strength with an increase in the number of seeds.

**Figure 4:**
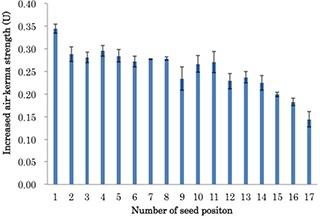
The increase in mean measured source strength with the addition of each seed. The positions of the seeds were numbered such that the seed at the bottom of the cartridge was labeled No. 1.

The reproducibility of measurements for each seed was found to be 1.1% (range: 0.1% to 3.8%). The reproducibility of measurements in the five‐seed cartridge and in the 15‐seed cartridge was found to be 1.2% and 0.2%, respectively. Analysis of the correlation between the number of seeds and the mean source strength yielded a correlation coefficient of 0.999 for a third‐order polynomial fit (Fig. 3). Despite this strong correlation, the rate of increase in source strength varied according to the source position in the cartridge (Fig. 4). The average (and standard deviation) of the increase in measured source strength in seeds No. 1 and No. 17 were 0.345 U±0.010 and 0.144 U±0.012, respectively.

### C. Correlation coefficients for single‐seed and batch assays

**Figure 5:**
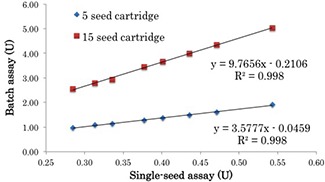
Plot showing the source strength as determined by single‐seed and batch assay for various seeds. Eight different source strength seeds (source strength: 0.539, 0.457, 0.429, 0.391, 0.364, 0.333, 0.306, and 0.282 U) were used.

The measured source strength for various single seeds is plotted in Fig. 5. A correlation coefficient of 0.998 was determined between the batch and single‐seed assay results for both the five‐seed and 15‐seed cartridges. The mean difference between the predicted source strength of the batch assay, as calculated using the regression formula, and the measured source strength of the five‐seed cartridge was found to be 0% (range: −0.1% to 1.6%). In contrast, the 15‐seed cartridge was found to be 0% (range: −2.6% to 1.5%).

### D. Impact of an aberrant seed on the batch assay

**Figure 6:**
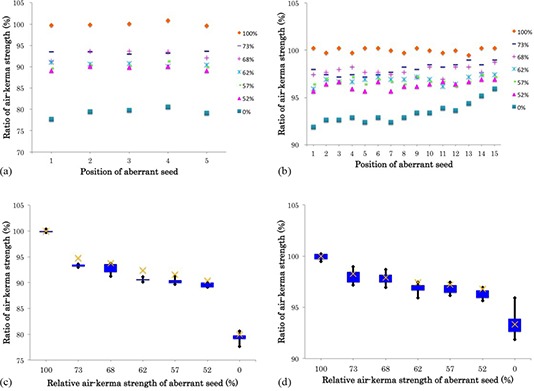
Plots showing the change in source strength when one seed was replaced with an aberrant seed in (a) the five‐seed cartridge and (b) the 15‐seed cartridge, and the change in the measured source strength according to the position of the aberrant seed in (c) the five‐seed cartridge and (d) the 15‐seed cartridge. On the vertical axis, 100% refers to the mean relative source strength in the case when all the seeds in a cartridge were reference seeds. The 60% quintile range and the range of data are displayed. The cross marker shows the theoretical source strength. The theoretical source strength was calculated using the following equation: (A×(N−1)+B)×100/A×N, where *A* is the mean source strength of the reference seeds, *B* is the mean source strength of each aberrant seed, and *N* is the number of seeds loaded in a cartridge (five or 15).

Figure 6 shows the change in source strength when one seed was replaced with an aberrant seed. The mean differences between the theoretical source strength and the mean measured source strength of the five‐seed and 15‐seed cartridges were found to be −1.2% (range: −0.7% to 1.8%) and −0.3% (range: 0% to 0.6%), respectively. Figures 6(a) and 6(b) show the change in the source strength measured with a change in the position of an aberrant seed; the response of the well chamber was found to be sensitive to the position of the aberrant seed when using the batch assay method. The asymmetry in the 15‐seed cartridge results may be due to the fact that the top of the cartridge is covered with brass and the bottom with polyethersulfone. Figures 6(c) and 6(d) change the x‐axis of the data of 6(a) and 6(b) into the relative air‐kerma strength of the aberrant seed. The 60% quintile range and the range of data are displayed, and the cross marker shows the theoretical source strength.

### E. Clinical quality assurance tests

**Figure 7:**
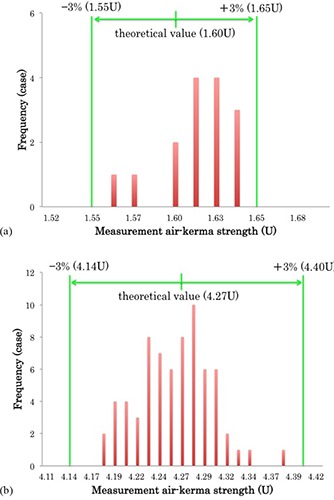
Frequency histogram of the measured source strength in (a) the five‐seed cartridge and (b) the 15‐seed cartridge.

The predicted source strength from the batch assay of the five‐seed and 15‐seed cartridges, as calculated using the formula of the approximated curve, was found to be 1.60 U and 4.27 U, respectively. Figure 7 shows the measured source strength for the five‐seed and 15‐seed cartridges sealed within an SCBP. The mean difference between the predicted source strength and the measured source strength in the five‐seed and 15‐seed cartridges was found to be 0.6% (range: −2.9% to 2.0%) and −0.6% (range: −2.1% to 2.7%), respectively.

## IV. DISCUSSION

In this study, a novel jig and a measurement method using a well ionization chamber were developed. This allowed for a batch assay characterization of the total source strength of I‐125 seeds within a cartridge sealed within an SCBP. To the best of our knowledge, this study is the first to describe the use of a batch assay to measure the source strength of I‐125 seeds with an SCBP. The method can be performed simply and quickly. However, to quantify the performance of the cartridge inspection schemes, Brame et al.^(^ ^12^ ^)^ demonstrated that in an assay using a shielded 15‐seed cartridge, the probability of detecting a missing or extra seed (i.e., a cartridge containing 14 or 16 seeds) is higher than for a single‐seed assay, or 10% of the seeds in a shipment.

The AAPM Low Energy Brachytherapy Source Calibration Working Group recommends that institutional medical physicists determine a source strength against which the measured source strength is evaluated when performing a batch assay. If a difference of more than 3% is found between the measured source strength of each cartridge and the reference value, the Working Group recommends the discrepancy be investigated. If a difference of more than 5% is found, consulting the manufacturer to resolve the difference is recommended.^(^ ^14^ ^)^ None of the cases investigated in this study exceeded the 3% deviation from the reference value that is generally tolerated, using our batch assay on an intact SCBP (Fig. 7). However, the calibration uncertainty of the well ionization chamber used in this study is 6% (k=2), so the difference from the normal value could exceed 5% even in normal cases, and even an abnormal reading may still be within the acceptable range. The AAPM TG138 indicates that in the calibration of a well ionization chamber by the University of Wisconsin Accredited Dosimetry Calibration Laboratory (ADCL), the propagation of best practice uncertainty is 2.56%.^(^ ^15^ ^)^ In other words, if the calibration uncertainty of a well ionization chamber becomes small, the reliability of this quality assurance method will improve.

Figure 6 shows the change in the measured source strength when one seed is exchanged with an aberrant seed. The aberrant seed in the five‐seed cartridge was easily detected when performing a batch assay. Nevertheless, the measurement method used in this study may fail to identify the aberrant seed because it cannot measure the source strength of each seed individually. For example, although the seed in position No. 15 in the 15‐seed cartridge was dead, the measured source strength was found to have decreased by only approximately 4% (Fig. 6(b)). This finding suggests that the use of an unshielded cartridge would likely allow for improved detection.

Figure 4 shows the rate of increase in source strength according to the source position in the cartridge. The rate of increase for the measurements was high in the No. 1 position and large standard deviations were observed in positions 9–12. The position of No. 1 had a hole in the cartridge and there was no absorption by another source. The position of Nos. 9–12 was close to the SCBP holding portion of the jig, which would have influenced absorption by the jig.

In this study, seeds of a higher activity than the standardized source strength were not used as aberrant seeds. However, Figs. 6(c) and 6(d) indicate that measured source strength was changed by aberrant seed activity and that we took the theoretical value for five‐seed cartridges. Therefore, even if higher activity aberrant seeds are used, they will follow a theoretical value.

Among the authors who have investigated different batch assay techniques, Lee et al.^(^ ^8^ ^)^ recommended the use of a five‐seed batch assay method with a plastic spacing holder and a well ionization chamber. Their study revealed that when exchanging a live seed with a dead seed, the measured source strength decreased by 20% with respect to the theoretical source strength. As shown in Figs. 6(a) and 6(c), the current study obtained similar results. Therefore, it can be assumed that our batch assay method has an equivalent dead seed location ability, even for seeds that are within the cartridge sealed in the SCBP.

Furutani et al.^(^ ^11^ ^)^ proposed the use of an imaging‐plate dosimetry system, in which they found a 0.999 correlation between the linear response of the source strength for a 15‐seed cartridge and the overall source strength. Use of this method can allow for the simultaneous characterization of 100% of seeds in a sterile environment, but requires that sterile inserts be deployed in the operating room relatively close to the time of the implant.

The results of the study by Furutani and colleagues indicate that the method most likely to detect aberrant seeds in a time‐efficient manner is one in which every cartridge in a shipment is measured, as does the method used in this study. Specifically, the method used in our study does not need to be performed in the operating room, poses no time limitations, and does not require resterilization. However, use of this method may pose a time penalty when the cartridge well chamber reading is out of the acceptance range, as the cartridge must be opened and the seeds individually assayed if this is the case. Despite this, our method offers additional advantages including the decreased probability of seed loss and minimization of personal exposure to radiation.

## V. CONCLUSIONS

We developed a novel jig for exclusive use with SCBP and carried out batch assays of source strength by using a well ionization chamber. This method is practical for all institutions needing to assay OncoSeed model 6711 seeds contained in an SCBP.

## ACKNOWLEDGMENTS

This study was partly supported by a Grant‐in‐Aid for Cancer Research from the Ministry of Health, Labour and Welfare of the Government of Japan (21‐8‐2).
